# National Vaccine Coverage Survey 2020: methods and operational aspects

**DOI:** 10.1590/1980-549720230031

**Published:** 2023-06-23

**Authors:** Rita Barradas Barata, Ana Paula França, Ione Aquemi Guibu, Maurício Teixeira Leite de Vasconcellos, José Cássio de Moraes, Grupo ICV 2020, Maria da Gloria Lima Cruz Teixeira, Carla Magda Alan Domingues, Maria Fernanda de Souza Oliveira Borges, Roberta Nogueira Calandrini de Azevedo, Consuelo Silva de Oliveira, Andrea de Nazaré Marvão Oliveira, Ivy Thereza Canales, Valdir Nascimento, Rejane Christine de Souza Queiroz, Luísa Helena de Oliveira Lima, Alberto Novaes Ramos, Jaqueline Caracas Barbosa, Isabelle Ribeiro Barbosa Mirabal, Meiruska Meira, Maria Bernadete de Cerqueira Antunes, Maria Denise de Castro Teixeira, Ricardo Queiroz Gurgel, Martha Suely Itaparica de Carvalho, Tayñana Cesar, Ethel Leonor Noia Maciel, Silvana Granado Nogueira da Gama, Karin Regina Luhm, Antônio Fernando Boing, Sotero Serrate Mengue, Sandra Maria do Valle Leone de Oliveira, Jaqueline Costa Lima, Sheila Araújo Teles, Karlla Antonieta Amorim Caetano, Wildo Navegantes de Araújo

**Affiliations:** I Santa Casa de São Paulo, Faculdade de Ciências Médicas – São Paulo (SP), Brazil.; II Sociedade para o Desenvolvimento de Pesquisa – Rio de Janeiro (RJ), Brazil.

**Keywords:** Vaccine coverage, Social inequalities, Birth cohort study, Population surveys, Coberturas vacinais, Desigualdades sociais, Estudo de coortes de nascimento, Inquéritos populacionais

## Abstract

**Objective:**

The national vaccination coverage survey on full vaccination at 12 and 24 months of age was carried out to investigate drops in coverage as of 2016.

**Methods:**

A sample of 37,836 live births from the 2017 or 2018 cohorts living in capital cities, the Federal District, and 12 inner cities with 100 thousand inhabitants were followed for the first 24 months through vaccine record cards. Census tracts stratified according to socioeconomic levels had the same number of children included in each stratum. Coverage for each vaccine, full vaccination at 12 and 24 months and number of doses administered, valid and timely, were calculated. Family, maternal and child factors associated with coverage were surveyed. The reasons for not vaccinating analyzed were: medical contraindications, access difficulties, problems with the program, and vaccine hesitancy.

**Results:**

Preliminary results showed that less than 1% of children were not vaccinated, full coverage was less than 75% at all capitals and the Federal District, vaccines requiring more than one dose progressively lost coverage, and there were inequalities among socioeconomic strata, favorable to the highest level in some cities and to the lowest in others.

**Conclusion:**

There was an actual reduction in full vaccination in all capitals and the Federal District for children born in 2017 and 2018, showing a deteriorating implementation of the National Immunization Program from 2017 to 2019. The survey did not measure the impacts of the COVID-19 pandemic, which may have further reduced vaccination coverage.

## RESUMO

**Objetivo:**

Inquérito nacional de cobertura vacinal aos 12 e 24 meses de idade foi realizado para investigar as quedas nas coberturas a partir de 2016.

**Métodos:**

Amostra de 37.836 nascidos vivos das coortes de 2017 e 2018 residentes nas capitais, Distrito Federal (DF) e 12 cidades com mais de 100 mil habitantes, acompanhados nos primeiros 24 meses por registros nas cadernetas de vacinação. Setores censitários foram estratificados segundo condições socioeconômicas, e o mesmo número de crianças foi incluído para cada estrato. Calcularem-se coberturas vacinais de cada vacina e coberturas completas aos 12 e 24 meses, doses aplicadas, válidas e oportunas. Fatores familiares, maternos e da criança associados à cobertura foram pesquisados. Os motivos para não vacinar analisados foram: contraindicações médicas, dificuldades de acesso, problemas no funcionamento do programa e hesitação vacinal.

**Resultados:**

Os resultados preliminares mostram que menos de 1% das crianças não foram vacinadas, as coberturas pelo esquema completo são menores que 75% em todas as capitais e no DF, as vacinas com mais de uma dose perdem cobertura progressivamente, há diferenças entre os estratos socioeconômicos, favoráveis aos estratos mais altos em algumas cidades e aos estratos mais baixos em outras.

**Conclusão:**

Houve realmente redução da cobertura vacinal em todas as capitais e no DF para as crianças nascidas em 2017 e 2018, denotando piora na execução do Programa Nacional de Imunizações durante os anos de 2017 a 2019. O inquérito realizado não mensurou os impactos da pandemia de COVID-19 que podem ter reduzido ainda mais as coberturas vacinais.

## INTRODUCTION

Routine vaccines of the National Immunization Program (*Programa Nacional de Imunizações* — PNI) are one of the public health interventions with the best cost-effective ratio regarding individual and collective benefits^1^. The population benefits depend on several factors, such as: reaching high coverage so that community immunity can curb the dissemination of the etiological agents under focus; attaining an even coverage distribution to avoid unprotected pockets; providing high quality of supplies and application procedures; providing access to services and abolishing socioeconomic and cultural barriers^2^.

Although the PNI was designed as a program to be universally provided for free, an uneven coverage has been reported for the first two years of life, depending on the vaccine, region, city, or socioeconomic status. Moreover, full vaccine coverage has remained below the recommended safety level^3–8^.

Vaccination coverage in Brazil has been dropping since 2016, raising issues such as: problems in records of doses administered after the implementation of the new information system (SI-PNI), outdated population estimates, the impact of misinformation and anti-vaccine activism, precariousness of primary care services due to underfunding of the Unified Health System (*Sistema Único de Saúde* — SUS), worsening of socioeconomic status, and vaccine hesitancy^7,9–13^.

In view of the persistent drop in vaccine coverage and lack of plausible explanations, the Department of Science and Technology of the Ministry of Health (*Departamento de Ciência e Tecnologia do Ministério da Saúde* — DECIT) commissioned a national vaccine coverage survey, whose methodology will be detailed in the present article.

The overall objectives of the survey were to: calculate vaccination coverage for each vaccine and full schedule for all children of the sample and per socioeconomic level; identify the factors associated with incomplete or no vaccination; and detect the reasons for difficult access, malfunctioning of the national immunization program, and issues related to vaccination hesitancy, defined herein as the views and behaviors of parents.

## METHODS

### Operational procedures

#### Calculation of sample size

The following formula was used to calculate the sample size for each survey:


$$\text{n}=[{\text{EDFF}}^{\text{*}}\text{Np}(\text{1-p})]\text{/}[({\text{d}}^{2}{\text{/Z}}_{1\text{-α/}2}^{2}\text{*}(\text{N-}1)+{\text{p}}^{\text{*}}(1\text{-p})]$$

where EDFF = the design effect by using census tract conglomerates, set at 1.4, based on previous studies^14^; N=hypothetical population of 1,000,000 live births (LB); estimated prevalence of vaccination coverage at 70%, d=estimation error=5%; z=1.96 for 95% confidence interval; resulting in n=452 children per survey.

#### Number of surveys and participants

Although a household survey was carried out, a historic cohort design was chosen, including children liveborn in 2017 or 2018, living in urban areas of each city, and that at the beginning of the study were 19 to 54 months old. Vaccine administration dates from birth throughout the initial 24 months of life were obtained.

The survey was carried out in 26 state capital cities, the Federal District (DF) and 12 additional cities with over 100 thousand inhabitants, located outside the metropolitan regions of capitals, in 12 states of the federation of different geographical regions — except for the North, where only capital cities were surveyed. Fieldwork was carried out between September 2020 and March 2022, taking into account the periods of social distancing applied in each location.

There were one to four surveys in each city, previously defined, depending on the number of LB registered on the 2017 and 2018 Liveborn Information System (*Sistema de Informações sobre Nascidos Vivos* — SINASC). Therefore, only one survey was performed at 15 cities (four capitals and 11 inner cities), two surveys were conducted in nine capitals, three surveys in four capitals, and four surveys in nine capitals, the DF and one interior city, totaling 89 surveys.

In order to enable comparison between the different socioeconomic strata, the sample size was divided to guarantee an approximately equal number of children in each socioeconomic stratum. Thus, for cities where only one survey was carried out, each stratum would include 113 children, 226 children for cities with two surveys, 339 children for cities with three surveys, and 452 children for those with four surveys, totaling 39,776 potential children.

### Sampling procedure

All urban census tracts of each city were used to define socioeconomic status, according to 2010 demographic census data. Data used to classify tracts were: mean income of head of household, number of literate household heads, and number of household heads with income equal to or above 20 minimum wages. Tracts were grouped by cluster analysis, using Euclidean distance, and results were adjusted to define four strata with at least the minimum number of children born in 2017 or 2018 required to attain the estimated sample size (STATA version 13).

Once census tracts were identified in each socioeconomic stratum, cohorts of children of interest living in each sector were estimated based on georeferencing of addresses in the SINASC and on the projection based on the distribution observed in the 2010 census.

In order to advance field work and assure spatial representativeness for each stratum, tracts were grouped by proximity and number of children expected, so that each cluster would have roughly three times the number of children to be included in the sample, to make up for possible address mistakes, changes, or other losses. A systematic draw resulted in clusters that covered the entire geographical area.

After attaining the maps of the drawn clusters and the list of addresses obtained from SINASC, the interviewers covered the area to locate the cohort children until reaching the pre-established number for each stratum and for each city (Table 1).

**Table 1:** Distribution of surveys, children per stratum, interviews performed and losses, in capitals and the Federal District, 2020–2021.

City	Surveys	Sample	Children per stratum	Children included	Losses(%)
Porto Velho	1	452	113	451	0.22
Rio Branco	1	452	113	451	0.22
Boa Vista	1	452	113	395	12.61
Palmas	1	452	113	452	0.00
Macapá	2	904	226	878	2.88
São Luís	2	904	226	878	5.53
Teresina	2	904	226	899	0.55
Natal	2	904	226	687	24.00
João Pessoa	2	904	226	904	0.00
Maceió	2	904	226	930	0.00
Aracaju	2	904	226	901	0.33
Vitória	2	904	226	788	12.83
Florianópolis	2	904	226	739	18.25
Cuiabá	2	904	226	815	9.85
Belém	3	1,356	339	1,219	10.10
Porto Alegre	3	1,356	339	1,383	0.00
Campo Grande	3	1,356	339	1,283	5.38
Manaus	4	1,808	452	1,827	0.00
Fortaleza	4	1,808	452	1,614	10.73
Recife	4	1,808	452	1,691	6.47
Salvador	4	1,808	452	1,819	0.00
Belo Horizonte	4	1,808	452	1,865	0.00
Rio de Janeiro	4	1,808	452	1,821	0.00
São Paulo	4	1,808	452	1,540	14.82
Curitiba	4	1,808	452	1,194	33.96
Goiânia	4	1,808	452	1,818	0.00
Brasília	4	1,808	452	1,809	0.00
Total	39	33,032		31,051	5.99

### Follow-up and losses

Losses were due to different factors, including refusals, impossibility of performing the interview after three attempts on different times and days of locating the expected number of children after an active search throughout the area of the drawn clusters (Table 1). Losses were 6% of the total sample, although there was a wide variation, with 19 cities without losses, up to 24% losses in the city of Natal, and 34% in Curitiba. Another six capitals had losses between 10 and 18% (Boa Vista, Belém, Fortaleza, Vitória, São Paulo and Florianópolis).

Regarding socioeconomic status, there were 18.8% losses in capitals and the DF, and 3.8% in interior towns for stratum A. For stratum B, corresponding findings were 6.4 and 0.4%. There were no losses for stratum C. Finally, for stratum D there were no losses in capitals nor in the DF, and there was a 0.1% loss in interior towns.

Like previous surveys, losses for stratum A were higher due to refusal to participate and inability to trace the children estimated. Similar difficulties were observed for stratum B in some cities^3,15,16^.

The longitudinal follow-up of children was based on the vaccine administration dates registered on vaccine record cards. Children without a card were considered as not vaccinated, after a manual data search on SI-PNI confirmed no administration of vaccine. When data were available on the SI-PNI, they were included in the survey database. The search was performed by the child's name, date of birth and mother's name.

As to the total sample, 99.2% had a vaccine card, with a small difference among social strata: 99.4% in stratum A; 99.1% in strata B and C; and 99.0% in stratum D.

### Collection tools

Two collection tools were used: a questionnaire and a photo of the child's vaccine card. A portable device electronic application was used to fill out questionnaires. The questionnaire comprised different sets of questions: the child's sociodemographic data; the mother's reproductive and sociodemographic data; household and family consumption data^17^; the child's vaccination data, reasons that led parents not to vaccinate, difficulties preventing vaccination, reason for not having vaccinated despite having sought services, guardians’ views on vaccines; vaccine card data (vaccines administered with date of administration).

### Team of interviewers, supervisors, and coordinators

Field work was performed by a company with expertise in scientific population-based surveys, that selected and trained interviewers and supervised field work. For each city, a coordinator was appointed by the national coordination to convey survey information to the local team, to support field team, and to deal with and solve possible issues.

Nurses trained in PNI activities read the data registered on vaccine cards and transferred data to the survey database.

### Consistency and quality control

In order to calculate coverages correctly considering the vaccines given by public or private services, the national coordination team was responsible for analyzing data consistency and for developing variables derived from registered data such as composite indicators, and for the rational ordering of dates to classify valid and timely doses for each vaccine, by joining different vaccines that prevent the same diseases (diphtheria-pertussis-tetanus — DTaP, tetra, pentavalent or hexavalent, for example).

### Classification of variables and concepts used

Household crowding was defined when more than three residents used the same room as a bedroom. The classification of the other variables is presented in results.

Level of family consumption was defined according to ABEP's Brazilian criterion thresholds^17^: high (42 points and above), medium (27 to 41 points), low (16 to 26 points) and very low (<16 points).

The variables related to parents’ views on vaccines are presented on the Likert scale and categories have been regrouped according to three possibilities for each statement presented: totally or partially agree, indifferent, totally or partially disagree.

**Figure 1:**
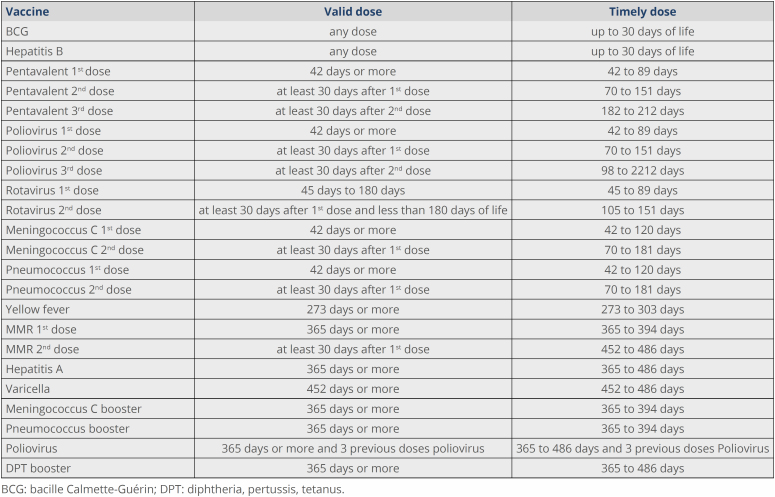
Criteria for defining valid doses and timely doses.

Doses were classified as valid and timely according to the time when they were administered, in relation to date of birth and intervals between doses (Figure 1).

Full basic vaccination included the vaccines that should have been given during the first year of life: bacille Calmette-Guérin (BCG), hepatitis B, three doses of pentavalent and polio vaccine, two doses of rotavirus, meningococcus C and pneumococcus, and one dose of yellow fever.

Full vaccination at 24 months of age included, in addition to the basic vaccine schedule, two doses of measles, mumps and rubella (MMR) vaccine, one dose of hepatitis A, varicella, and polio, and one booster dose of DTaP, meningococcus C and pneumococcus. Vaccine schedules were calculated for total valid doses and timely doses.

### Statistical analysis

Given the sample was stratified and clustered by census sector, and disproportionate allocation strategies (DAS) were used, it was necessary to calculate and use sample weights for each household interviewed, to enable unbiased estimation of the parameters of interest in the population.

Sample weights were calculated in two steps. First, we obtained the basic sample weights, corresponding to the inverse of the inclusion probabilities of the households interviewed. Weights were then calibrated to known population totals to correct any distortions in the sample distribution, due to a possible differential nonresponse or if the sample could possibly represent the population of households without considering breakdown by sex and age of their residents.

Certainly, household samples tend to be biased when estimating population by sex and age, simply because it is impossible to control composition by sex and age of residents beforehand, which requires calibrating weights to correct such biases.

Essentially, calibration estimates factors (named calibration factors) that multiply basic weights to generate calibrated weights, that minimize differences between estimates obtained from the basic sample weights and the corresponding population totals (known through other sources) for a set of auxiliary calibration variables or post-strata^18^.

The most usual method for calibrating household sample weights is the integrated household weighing system, which assigns a calibrated weight to each household, calculated to minimize differences between the estimates obtained by basic weights and the totals of household populations and individuals obtained from a source external to the survey^19^.

However, in the present survey, the relevant data are children's vaccination history and the possible reasons that led their guardians not to vaccinate them, which allowed simplifying the calibration procedure. A post-stratification calibration procedure was performed, with post-stratum defined by the domain. The total population of each domain refers to the 3- and 4-year-old urban population for June 1, 2021, obtained using the linear trend method, the same the Brazilian Institute of Geography and Statistics (*Instituto Brasileiro de Geografia e Estatística* — IBGE) uses in its population projections^20,21^. For further reference, more details can be requested from the authors regarding probabilistic sampling techniques used in the survey.

Point estimates of totals, means, percentiles, etc., can be obtained by using children's calibrated weights alone. For variance estimates and its dependent measures (such as standard deviation, coefficient of variation, half-width of confidence intervals, p-value, etc.), there are three sources of variability:

1. Sampling;
2. Using a complex sample; and
3. Calibration process.

Given calibration was post-stratification, STATA can deal with the three sources of variability if the structural information of the sampling plan is defined: basic weight, primary sampling unit, selection stratum and calibrated weight, all information included in the survey database.

All point estimates and their confidence intervals were calculated using the STATA version 17 survey data analysis module, considering the sources of variability mentioned above.

### Ethical aspects

The survey project was approved by the Human Research Ethics Committee of Irmandade da Santa Casa de São Paulo.

Before starting the study, local coordinators contacted the individuals responsible for state and municipal immunization programs in each city to inform them about the survey.

Interviewees signed a consent form for the interview and for having their vaccine record cards photographed. Data were only collected after guardian consent.

The database for processing was set up without the identification data of interviewees, thus avoiding breach of survey participant confidentiality.

After preliminary processing, results were presented to the individuals responsible for the state and municipal immunization programs, along with the local coordinator and a member of the national coordination of the survey, and enabled presenting the scenario for each municipality and discussing strategies to improve the coverages observed.

## RESULTS

Preliminary data refer only to capitals and the DF. Less than 1% of the total sample had not received any vaccine dose. Twenty-three percent of guardians stated having used a private service for application of at least one vaccine, dropping from 55.7% in stratum A to 9.0% in stratum D.

Table 2 shows the characteristics of the sample studied according to the socioeconomic strata of residence areas (census tracts). These characteristics are related to the families, mothers, and children included in the sample.

**Table 2:** Sociodemographic characteristics of families, mothers and children included in the survey in all capitals and the Federal District, 2020–2021.

Variable		Stratum A	Stratum B	Stratum C	Stratum D	Total
N. families included		6,695	7,724	8,280	8,329	31,028
Characteristics of families						
Level of family consumption (ABEP) – % families						
	High	20.7	10.0	3.3	0.5	4.5
	Medium	51.6	41.9	33.5	8.8	22.6
	Low	17.1	30.4	34.8	38.8	34.5
	Very low	10.7	17.7	28.5	52.0	38.5
Household crowding (%)		3.7	5.5	8.6	14.8	11.2
*Bolsa Família Program* (%)		7.3	12.5	19.3	34.4	25.6
Monthly family income (%)						
	Up to R$ 1,000	5.4	10.8	18.5	37.3	26.7
	R$ 1,001 to R$ 3,000	10.9	20.2	31.3	41.6	33.5
	R$ 3,001 to R$ 8,000	23.3	25.1	23.2	9.7	15.8
	Above R$ 8,000	37.3	20.1	10.3	1.2	9.5
Grandmother living in household (%)		23.3	23.0	27.6	30.6	28.2
Mother's characteristics						
Schooling (%)						
	0 to 8 years	1.6	4.0	5.5	11.6	8.3
	9 to 12 years	4.0	7.0	10.2	21.8	15.7
	13 to 15 years	22.1	26.8	39.5	48.2	40.9
	16 years and more	70.3	59.8	41.8	15.4	32.3
Age group						
	<20 years	0.9	1.4	1.7	3.2	2.4
	20 to 34 years	38.2	40.8	52.9	62.0	54.9
	35 years and more	60.6	57.3	45.2	34.4	42.3
Race/color (%)						
	White	58.3	57.5	51.3	36.5	44.5
	Brown	32.0	30.8	36.7	42.7	38.8
	Black	6.9	7.0	7.9	16.9	12.7
	Yellow	1.2	2.5	1.2	1.3	1.4
	Indigenous	0.0	0.3	0.4	0.3	0.3
Paid work (%)		70.8	63.1	57.0	46.3	53.3
With partner (%)		87.1	82.4	75.8	69.3	74.2
Number of live children (mean)		1.9	1.8	1.9	2.3	2.1
Characteristics of children						
Sex						
	Male	48.5	47.9	51.3	51.8	50.8
	Female	51.5	52.1	48.7	48.3	49.2
Birth order						
	First-born	51.9	54.9	52.4	43.7	47.7
	Second-born	36.1	30.4	32.1	29.6	30.9
	Third-born	8.4	10.2	10.6	15.1	12.8
	Fourth-born or more	3.4	4.4	4.8	11.4	8.3
Race/color						
	White	69.1	63.4	59.9	44.9	52.9
	Brown	26.9	30.8	34.4	43.0	37.9
	Black	2.6	4.5	4.6	11.1	8.0
	Yellow	1.3	0.9	0.7	0.8	0.9
	Indigenous	0.0	0.0	0.3	0.3	0.3
Child in daycare		57.7	53.5	48.3	48.0	49.8

ABEP: Associação Brasileira de Empresas de Pesquisa.

Even in the high socioeconomic stratum (A), only 20% of families had a high level of consumption, and most had a medium level, while in the very low stratum (D) only 0.5% had high-level consumption, and most had a very low consumption level. For the total sample, less than 5% of families had high level consumption, roughly 25% had medium or high consumption and the remaining 75% were divided between low or very low consumption. Only part of the families classified in the very low consumption bracket benefited from the cash transfer program (*Bolsa Família Program*), and most families stated a monthly income of up to three minimum wages.

Most mothers had more than 12 years of schooling, and there was a major inequality among socioeconomic strata. The majority of them were between 20 and 34 years old, but 60% of those in stratum A, and 57% of those in stratum B were 35 years old or older. Distribution of race/color was similar among strata, except for stratum D with a higher proportion of brown and black individuals. A little over half of the mothers referred paid work, at a percentage directly proportional to the socioeconomic strata. Almost 75% referred living with a partner, at a percentage directly proportional to the socioeconomic strata.

Most children in the sample were the first or second child, with less than 10% whose birth order was third or younger. Almost half of the children attended daycare centers, at a percentage directly proportional to the socioeconomic strata.

Table 3 shows the vaccine coverages, including administered doses for each of the vaccines scheduled for the first two years of life. The highest coverage was for the first dose of the pneumococcus vaccine, followed by the first dose of meningococcus C, surpassing the BCG, which usually has higher coverages. The lowest coverages were found for yellow fever, the second dose of rotavirus and MMR vaccines. There was a progressive drop in coverages between doses for vaccines requiring more than one dose, and likewise for booster doses.

**Table 3:** Vaccine coverage (doses given) for each vaccine of the schedule for first two years of life, birth cohorts 2017 and 2018, capitals and the Federal District.

Vaccine	Coverage (95%CI)	Vaccine	Coverage (95%CI)
BCG	89.6 (88.4–90.78)	Meningococcus C 1^st^ dose	92.0 (90.9–92.9)
Hepatitis B	88.7 (87.4–89.9)	Meningococcus C 2^nd^ dose	89.3 (88.1–90.3)
Pentavalent 1^st^ dose	91.6 (90.5–92.6)	Yellow fever	76.4 (74.4–78.3)
Pentavalent 2^nd^ dose	90.1 (89.0–91.2)	MMR 1^st^ dose	90.8 (89.7–91.8)
Pentavalent 3^rd^ dose	87.9 (86.6–89.1)	MMR 2^nd^ dose	82.0 (80.6–83.4)
Poliovirus 1^st^ dose	91.9 (90.9–92.9)	Hepatitis A	88.1 (86.8–89.2)
Poliovirus 2^nd^ dose	90.6 (89.4–91.7)	Varicella	86.9 (85.6–88.1)
Poliovirus 3^rd^ dose	87.8 (86.4–89.0)	Pneumococcus booster	84.8 (83.5–86.1)
Pneumococcus 1^st^ dose	92.3 (91.2–93.1)	Meningococcus C booster	82.0 (80.6–83.2)
Pneumococcus 2^nd^ dose	90.3 (89.1–91.3)	Poliovirus booster	86.0 (84.7–87 1)
Rotavirus 1^st^ dose	87.8 (86.4–88.8)	DPaT booster	83.9 (82.6–85.1)
Rotavirus 2^nd^ dose	82.0 (80.6–83.3)	Full schedule*	59.9 (58.3–61.5)

BCG: bacille Calmette-Guérin; DPaT: diphtheria, pertussis, tetanus.

* Yellow fever was not included because its introduction into the basic schedule varied among states and not all of them had implemented it by 2017.

Table 4 presents estimates and confidence intervals for the full schedule at 24 months of age, in a descending order, for each state capital and the DF, and the differences in coverage between high (A) and very low (D) socioeconomic strata. None of the cities studied had satisfactory coverages, all showing rates below 80%, even taking into account the doses administered regardless of their being valid or timely. Of the 27 cities studied, only three had coverages above 70%, ten were between 60 and 69%, ten between 50 and 59%, and four below 50%.

**Table 4:** Full coverage (doses given) and difference between coverages of socioeconomic stratum A and stratum D, for each capital and the Federal District, 2017 and 2018 cohorts.

City	Full coverage*	Inequality: difference betweenhigh and very low stratum (%)
Curitiba	74.4 (66.3–81.1)	-3.2
Teresina	73.7 (63.0–82.1)	10.4
Brasília	73.1 (69.3–76.5)	-0.4
Palmas	67.5 (60.5–73.8)	-8.7
Aracaju	65.3 (58.7–71.4)	2.9
Porto Alegre	65.2 (59.7–70.3)	-6.1
Salvador	65.0 (60.6–69.1)	-15.3
Porto Velho	64.8 (58.0–71.1)	18.2
São Paulo	64.0 (60.1–67.7)	-5.3
Belo Horizonte	63.8 (59.5–67.9)	-9.2
Cuiabá	60.9 (53.2–68.0)	16.1
Rio Branco	60.8 (53.3–67.7)	3.1
Boa Vista	60.0 (48.8–70.2)	14.3
Maceió	58.3 (50.2–66.0)	-4.5
Belém	57.5 (47.4–67.0)	7.8
Vitória	57.1 (50.6–63.6)	-33.8
Recife	56.9 (49.6–63.9)	-14.7
Goiânia	56.6 (50.2–62.8)	-8.8
Campo Grande	54.2 (48.2–60.0)	-12.4
Manaus	54.1 (49.6–58.6)	-3.7
Fortaleza	54.0 (47.3–60.5)	-24.1
São Luís	51.6 (43.1–60.1)	17.4
Rio de Janeiro	51.6 (45.8–57.4)	5.1
Florianópolis	49.6 (40.8–58.4)	-8.2
João Pessoa	42.6 (36.3–49.2)	-12.2
Natal	36.6 (26.8–47.8)	-24.4
Macapá	35.8 (28.1–44.3)	7.7

* Yellow fever was not included because its introduction into the basic schedule varied among states and not all of them had implemented it by 2017.

Social inequality is reflected in the unequal coverages reported for high and low socioeconomic strata as higher coverage was found for the high stratum in ten cities, with differences ranging from 2.9 to 18.2 percentage points, and lower coverage among the poorest for six cities of the Northeast, two of the North, one of the Southeast and one of the Center-West. Coverages for higher and lower strata were practically equivalent in the DF, and for the remaining cities studied coverages were lower in the higher stratum, with a difference between 3.2 and 33.8 negative percentage points, although all cities presented unsatisfactory coverages.

## DISCUSSION

Household surveys using vaccine record card data have major advantages such as: obtaining data regardless of flaws in registration systems; acquiring data from public and private services on non-vaccinated children; and collecting population data for accurate coverage calculation without the interference of population estimates^22,23^.

There are also, however, some drawbacks, mainly costs and time required for finishing the survey. The challenges faced in the present survey can be grouped into difficulties in access to families due to mistrust, urban insecurity, or lack of interest to take part in surveys; and difficulties in reading vaccine record cards, given there is no standardization among the models used, there are frequent non-legible registrations, and clear errors on registered dates. As the 2020 demographic census was not held, socioeconomic strata were defined using outdated data, hence comparisons may have been affected for cities where urban changes were more pronounced. Family status data can help identify such issues to a certain extent, given the limitation of the classification used^24^.

There is a relatively high proportion of mothers aged 35 years or older and having complete higher education in the total sample, which suggests the occurrence of selection bias. In view of the fact that the demographic census was not carried out in 2020, we had to use the 2010 data for the census tracts stratification. In several capitals, the characteristics of the sectors may have changed in this interval of ten to 12 years. Furthermore, the only data available on schooling for the census tracts was the proportion of functional illiteracy of those responsible for the families. In the preliminary analysis, maternal age was not shown to be related to levels of vaccination coverage, so that the bias observed in this aspect should not impact the results. Maternal schooling is directly associated with coverage levels, which implies an estimate of coverage that is probably overestimated.

Preliminary results allow us to state that there has been a major drop in vaccination coverage in the country, which cannot be explained only by the change in the information system. Coverages have dropped progressively for all vaccines requiring more than one dose; certain vaccines have lower coverages than others, such as rotavirus and MMR vaccines^25^; the COVID-19 pandemic certainly made routine vaccination difficult; and the decrease in vaccine coverage is multifactorial.

Further detailed analyses of data referring to access, functioning of the program, and hesitancy may provide some clues for understanding our findings, and thus guide PNI actions to attain higher coverages, similar to those reported in the past.
